# Characterizing the Gut Microbial Metabolic Profile of Mice with the Administration of Berry-Derived Cyanidin-3-Glucoside

**DOI:** 10.3390/metabo13070818

**Published:** 2023-07-04

**Authors:** Pengcheng Tu, Xiaodong Zheng, Huixia Niu, Zhijian Chen, Xiaofeng Wang, Lizhi Wu, Qiong Tang

**Affiliations:** 1 Department of Environmental Health, Zhejiang Provincial Center for Disease Control and Prevention, 3399 Binsheng Road, Hangzhou 310051, China; tupengcheng1@163.com (P.T.); niuhuixia1118@163.com (H.N.); zhjchen@cdc.zj.cn (Z.C.); xfwang@cdc.zj.cn (X.W.); 2 Department of Food Science and Nutrition, Zhejiang University, Hangzhou 310058, China; xdzhengzju2022@163.com; 3 College of Standardization, China Jiliang University, Hangzhou 310018, China

**Keywords:** cyanidin-3-glucoside, berries, gut microbiota, bacteria, metabolites

## Abstract

Dietary modulation of the gut microbiota has recently received considerable attention. It is well established that consumption of berries confers a number of health benefits. We previously reported that a black raspberry (BRB)-rich diet effectively modulates the gut microbiota. Given the role of anthocyanins in the health benefits of berries, coupled with interactions of gut microbial metabolites with host health, the objective of this follow-up study was to further characterize the profile of functional metabolites in the gut microbiome modulated by anthocyanins. We utilized a berry-derived classic anthocyanin, cyanidin-3-glucoside (C3G), combined with a mouse model to probe C3G-associated functional metabolic products of gut bacteria through a mass spectrometry-based metabolomic profiling technique. Results showed that C3G substantially changed the gut microbiota of mice, including its composition and metabolic profile. A distinct metabolic profile in addition to a variety of key microbiota-related metabolites was observed in C3G-treated mice. Microbial metabolites involved in protein digestion and absorption were differently abundant between C3G-treated and control mice, which may be linked to the effects of berry consumption. Results of the present study suggest the involvement of the gut microbiota in the health benefits of C3G, providing evidence connecting the gut microbiota with berry consumption and its beneficial effects.

## 1. Introduction

The key role of the gut microbiota has been increasingly recognized in recent years. Gut microbes are involved in host metabolic processes and physiological functions such as food digestion, energy production, immune development, epithelial homeostasis, and so forth [1,2,3,4]. Metabolic activities of the gut microbiota significantly contribute to host fitness. Gut bacteria communicate with the host though metabolites produced via a number of metabolic activities and interactions [5]. For instance, bile acids, which are cholesterol derivatives that can be modified by gut bacteria, can act as signaling molecules via the activation of nuclear receptors [6]. Likewise, short-chain fatty acids (SCFAs) are another important class of gut microbiota-derived metabolites produced by bacterial fermentation of dietary fiber, and serve as natural ligands for receptors across a range of cell and tissue types [7]. Thus, the gut microbiota, especially its metabolite profile, plays an essential role in host health and physiology.

Diet, the gut microbiota, and human health are profoundly intertwined. A Western diet high in fat and calories is associated with increased risks of multiple metabolic diseases, such as obesity, diabetes, cardiovascular disease, and chronic inflammation [8,9]. On the contrary, adding berries rich in polyphenols and soluble fiber to the diet can prevent such metabolic diseases [10,11]. Diet is an essential determinant of gut microbial structure and functions; meanwhile, the gut microbiota is closely related to host metabolism and health. Therefore, changes in the gut microbiota caused by distinct dietary components may contribute to their corresponding health effects. Dietary modulation of gut microbiota has the advantages of high safety and high patient compliance. However, effective approaches to modulating the gut microbiota still need to be explored.

Berries, including raspberries, blueberries, strawberries, and cherries, are fruit with diverse health benefits, including anti-inflammatory, antioxidant, and anti-carcinogenic effects [12,13,14,15]. We previously reported that consumption of black raspberries was associated with the effective modulation of the gut microbiome [16,17,18]. In our previous work, the composition and functions of the gut microbiota of healthy mice fed black raspberries (Rubus occidentalis) were characterized [16]. In addition to alterations at compositional and pathway levels, the gut microbiota of mice had unique metabolite profiles resulting from reprogrammed metabolic activities of black raspberry-type gut microbiota [17,18], which demonstrates the potential of black raspberries to modulate the gut microbiota.

Anthocyanins, abundant in berries, are natural pigments with outstanding antioxidant capability [19]. Moreover, anthocyanins are a key bioactive component in berries that confer health benefits [20,21]. To better understand the role of anthocyanins in the health benefits of berry consumption, in this follow-up study, we integrated metagenomic and metabolomic analyses to investigate the effects of a classic anthocyanin, cyanidin-3-glucoside, on the composition and functions of the gut microbiome. Results of the present study revealed a distinct metabolic profile in addition to a range of key microbiota-related metabolites, including microbial metabolites involved in protein digestion and absorption, which may be linked to beneficial effects of berry consumption. The findings of this study provided a better understanding of health benefits resulting from berry consumption through the lens of the gut microbiota and its functional metabolites.

## 2. Materials and Methods

### 2.1. C3G Preparation

Preparation of C3G was conducted as previously described [22]. Briefly, berry fruits (bayberries) were ground and extracted using a 4-fold volume solution of 100% ethanol containing 1% HCl for 24 h at 4 °C. Filtered fluid was evaporated at 49 °C, the concentrate was then loaded onto a D101 macroporous resin column, followed by elution with 1% formic acid in 80% methanol for further purification. Freeze-dried C3G powder was obtained and stored at −80 °C before use. An ultra high-performance liquid chromatography (UPLC) instrument (Thermo UltiMate 3000, Thermo Fisher Scientific, Waltham, MA, USA) was used to identify anthocyanin extract components and, based on the UPLC result shown in our previous report [23], the purity of C3G used in the present study was about 90.5% (905 mg/g).

### 2.2. Animals and Experimental Design

Twenty male C57BL/6 mice, approximately 4 weeks old and specific-pathogen-free (SPF), were procured from SLAC Laboratory Animal Co., Ltd., (Shanghai, China). Five mice were housed in a cage during acclimation. Following a week of acclimation, the mice were randomly divided into two groups: a control group (*n* = 10) and a cyanidin-3-glucoside (C3G) treatment group (*n* = 10). For randomization, for each group, 10 mice were therefore assigned into 3 cages (*n* = 3; *n* = 3; *n* = 4) to reduce the cage effect. The criterion of randomization was that each mouse in a new cage came from different cages during acclimation, meaning every single mouse in a new cage was never housed together before, ensuring random allocation.

Mice in the treatment group received a daily oral dose of C3G water solution (150 mg/kg/day) via gavage, based on previous studies [22,23,24,25,26,27], while control mice were administered an equivalent volume of water also via gavage. The mice were maintained under controlled conditions of 22 °C temperature, 40–70% humidity, and a 12/12 h light/dark cycle. After six weeks of C3G administration, individual fecal samples were collected, immediately snap-frozen in liquid nitrogen, and stored at −80 °C. Prior to euthanasia, mice were fasted for 12 h and then euthanized in a carbon dioxide chamber. All procedures were conducted in accordance with relevant guidelines and approved by the Animal Care and Use Committee of Zhejiang Chinese Medical University (No. 20201103-08).

### 2.3. 16S rRNA Gene Sequencing

Microbial DNA from fecal samples was extracted using the QIAamp DNA Stool Mini Kit (Qiagen, Germany) following the manufacturer’s instructions. The V4 region of the 16S rRNA gene was amplified using PCR with 515F and 806R primers. Purified amplicons were pooled equimolarly and subjected to paired-end sequencing on an Illumina MiSeq platform (Illumina, San Diego, CA, USA). Raw fastq files were quality-filtered using Trimmomatic and merged using FLASH. Operational taxonomic units (OTUs) were clustered with a 97% similarity cutoff using UPARSE (version 7.1). Taxonomic analysis of 16S rRNA gene sequences was conducted using the RDP classifier algorithm.

### 2.4. Metabolite Profiling Analysis

Metabolite extraction from mouse fecal samples (50 mg) was performed by mixing with a pre-cooled extraction solution (methanol:acetonitrile:water in a 2:2:1 ratio, *v*/*v*). An internal standard, L-2-chlorophenylalanine (20 μL), was added to the mixture. The samples were then subjected to ultrasonic extraction for 30 min at 5 °C. Following a 10-min incubation at −20 °C, the samples were centrifuged at 14,000× *g*/4 °C for 20 min. The supernatant was subsequently evaporated using nitrogen. The dried samples were reconstituted in 100 μL of acetonitrile solution (acetonitrile:water in a 1:1 ratio, *v*/*v*), vortexed for 30 s, and centrifuged at 14,000× *g*/4 °C for 15 min. The resulting supernatant was reserved for injection.

Fecal metabolite profiles were analyzed using an Agilent 1290 Infinity LC Ultra-High Performance Liquid Chromatography (UPLC) System coupled with a Thermo Q Exactive Mass Spectrometry (MS) System with a HILIC column at Shanghai Applied Protein Technology Co., Ltd., (Shanghai, China). A quality control sample was prepared by pooling 20 µL of supernatant from each sample.

Raw data, initially in Wiff format, were converted into mzXML format using ProteoWizard and processed with XCMS software for baseline filtering, peak identification, retention time correction, and peak alignment. Metabolite identification was conducted based on the accurate *m*/*z* value (<25 ppm) and comparison of MS/MS spectra with an in-house database established by Shanghai Applied Protein Technology Co., Ltd., (Shanghai, China), which was constructed using available authentic standards.

### 2.5. Statistical Analysis

Differences in gut microbial abundances were assessed by a nonparametric test using Metastats. Furthermore, linear discriminant effect size (LEfSe) analysis was used to identify differentially enriched bacteria. Principal coordinate analysis (PCoA) was used to assess diversities in the gut microbial communities. PLS-DA and volcano plots were used for visualization of the comparison of metabolite profiles. A two-tailed Welch’s *t*-test was used to analyze metabolites that were differently abundant between groups corrected for the FDR. Histograms were presented by GraphPad Prism (version 8.0.1). A value of *p* < 0.05 (adjusted for FDR) was considered to be a statistically significant difference between groups.

## 3. Results

### 3.1. Workflow to Probe Functional Alterations of the Gut Microbiota of Mice upon C3G Administration

**Figure 1:**
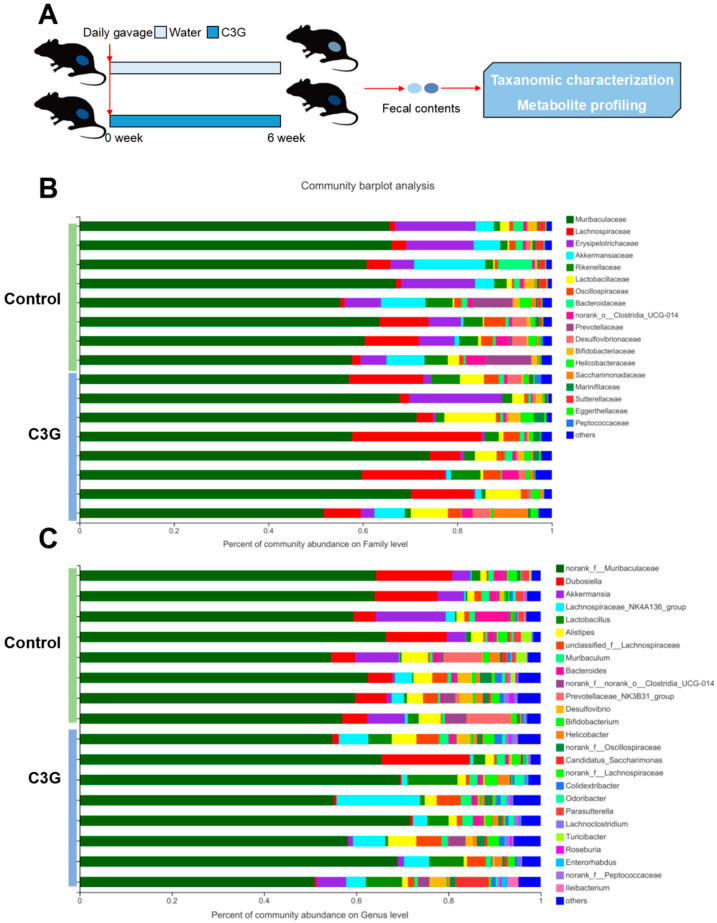
C3G-modulated gut microbiota composition. (**A**) Experimental design (*n* = 10). (**B**) Taxonomic summary at family level (*n* = 8). (**C**) Taxonomic summary at genus level (*n* = 8).

The experimental workflow is shown in Figure 1A. Briefly, mice were randomly assigned into two groups: control and C3G-treated groups. Mice of the C3G-treated group were administered with C3G daily via gavage, while controls were gavaged with an equivalent volume of water. After six weeks of treatment, fecal samples were individually collected for taxonomic characterization and metabolite profiling. The experimental workflow combined high-throughput 16S rRNA gene sequencing and mass spectrometry-based metabolomics for the examination of changes in the gut microbiota of mice resulting from C3G administration.

### 3.2. Gut Microbial Changes of Mice upon C3G Administration at Compositional Level

The overall composition of the gut microbiota community was examined by assessing taxonomic similarity between sequencing samples in different groups. Figure 1B,C shows the identified gut bacteria at family or genus levels assigned by 16S rRNA sequencing, with each color representing an individual bacterial family or genus, respectively. Particularly, according to the community barplot analysis at the family level (Figure 1B), the gut microbiota of C3G-treated mice differed from that of control mice. C3G treatment was associated with an increase in Lactobacillaceae and Lachnospiraceae and a reduction in Erysipelotrichaceae and Akkermansiaceae. The composition of the gut microbiota at the genus level was also analyzed (Figure 1C), which revealed differential bacterial genera between the control and C3G-treated groups. Consistently, C3G administration is associated with an increase in Lactobacillus and the Lachnospiraceae_NK4A136 group as well as a decrease in Dubosiella and Akkermansia. Taken together, C3G administration modulated the composition of the gut microbial community with populations of key bacterial families and genera being altered.

### 3.3. Comparative Analysis of the Gut Microbiota Communities

**Figure 2:**
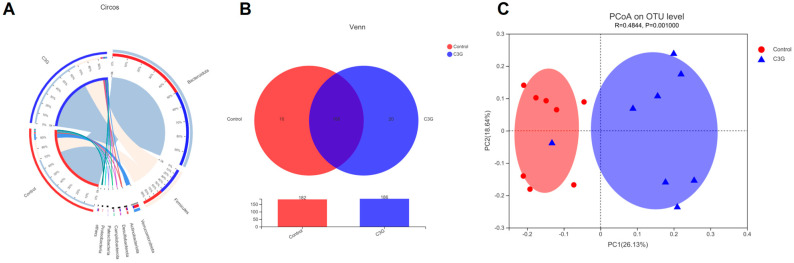
Comparative analysis of mouse gut microbial communities. (**A**) Circos diagram of the component profiles of the gut microbial community at the phylum level (*n* = 8). (**B**) Venn diagram of the component profiles of the gut microbial community at the species level (*n* = 8). (**C**) Component profiles analyzed by the PCoA model (*n* = 8).

**Figure 3:**
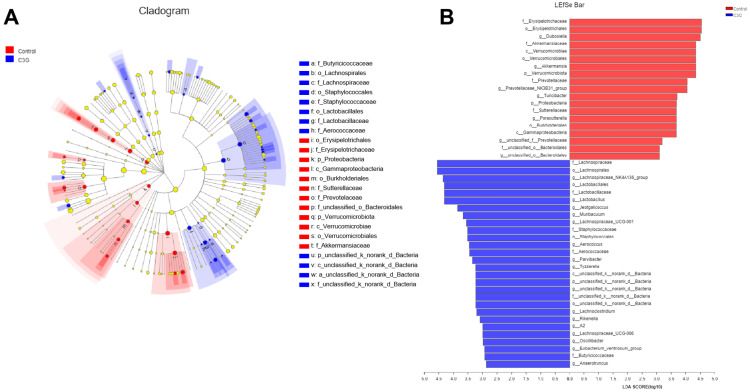
Comparison of gut microbiota by LEfSe analysis. (**A**) Cladogram (phylum to family) made through discriminant analysis of LEfSe (*n* = 8). (**B**) Discriminant analysis of LEfSe multi-level species differences (*n* = 8).

Figure 2A shows a comparison of proportions of main phyla in the gut microbiota. The most abundant phyla are Firmicutes and Bacteroidetes, followed by Verrucomicrobiota, together dominating more than 90 percent of the gut microbiota composition. The proportion of sequences assigned to Firmicutes was higher in fecal samples of mice administered with C3G, whereas sequences assigned to Bacteroidetes were close between different groups. The proportion of sequences assigned to Verrucomicrobiota was lower in the feces of C3G-treated mice compared to controls. Further analysis revealed that the C3G-treated and control groups shared 166 bacterial genera (Figure 2B). Interestingly, there were sixteen and twenty unique genera in the gut microbiotas of control mice and C3G-treated mice, respectively. Moreover, microbiota communities were clustered using principal coordinate analysis (PCoA), which disclosed that C3G-treated mouse gut microbiota were well separated from controls (Figure 2C). In addition, the cladogram also displayed gut bacterial differences between C3G-treated mice and controls (Figure 3A). LEfSe analysis disclosed that administration of C3G is associated with a reduction in the populations of Dubosiella (f_Erysipelotrichaceae) and Akkermansia (f_Akkermansiaceae), and an expansion in the populations of Lactobacillus (f_Lactobacillaceae) and the Lachnospiraceae_NK4A136 group (f_Lachnospiraceae) (Figure 3B), which is consistent with the results of our community barplot analysis. Taken together, the results of the comparative analysis further suggested that the gut microbiota community is different in mice upon administration of C3G, with differences at phylum, family, and genus levels.

### 3.4. Gut Microbial Changes of Mice upon C3G Administration at the Metabolite Level

**Figure 4:**
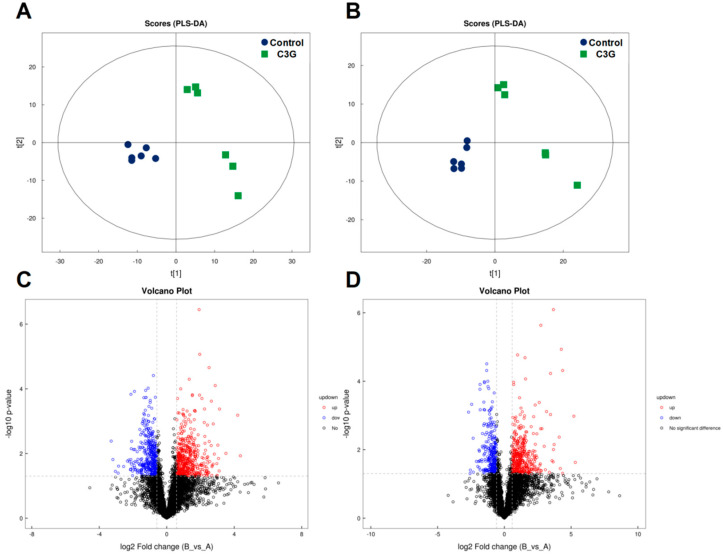
Comparisons of metabolite profiles (*n* = 6). PLS-DA score charts of metabolite profiles in positive (**A**) and negative (**B**) ion modes. Volcano plots represent distribution of metabolite fingerprints in positive (**C**) and negative (**D**) ion modes.

**Figure 5:**
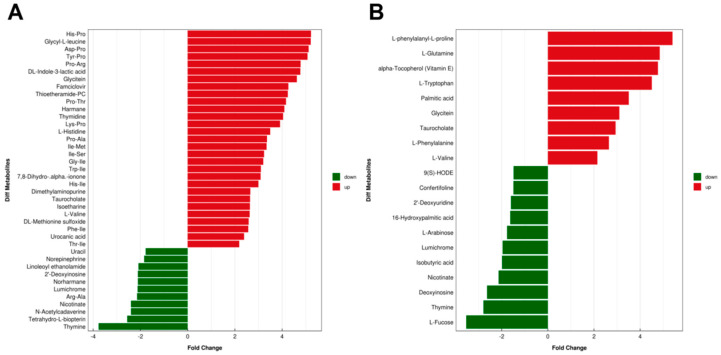
Identified metabolites in the gut microbiota associated with C3G administration. Fold change and classification of identified metabolites under positive (**A**) and negative (**B**) ion modes. (*n* = 6).

To assess the impact of C3G on metabolite profiles of the gut microbiota, non-targeted metabolomics was performed on mouse fecal samples. PLS-DA analysis clearly separated the two cultivars under both positive and negative ion mode conditions (Figure 4A,B). Differential metabolites were screened with the criteria of a fold change >1.5 or <0.67 and a *p*-value < 0.05, which was visually presented by volcano plots (Figure 4C,D). In addition, Figure 5A,B shows fold changes of identified metabolites that were significantly altered in fecal samples of mice upon C3G administration under positive and negative ion mode conditions, respectively. These C3G-altered fecal metabolites are diverse, with many of them associated with metabolic activities of the gut microbiome, meaning they possess key functions interacting with host, possibly conferring health effects (Table S1). Taken together, results of metabolite profiling indicated that C3G administration modulated metabolic profiles of the gut microbiota and also revealed a number of key metabolites associated with the administration of C3G, including bile acids, tocopherols, amino acids, and associated derivatives.

### 3.5. Enrichment Analysis of Metabolites Associated with C3G Administration

**Figure 6:**
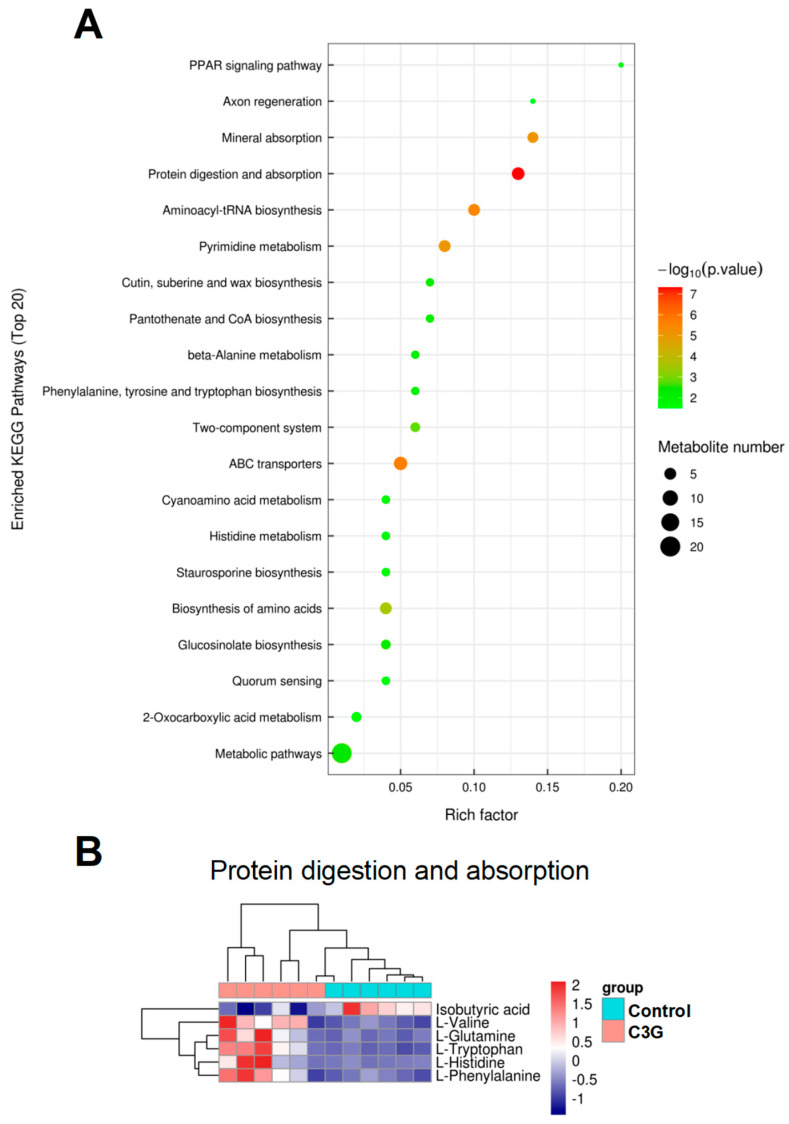
KEGG enrichment analysis. (**A**) The X-axis rich factor is the number of differential metabolites annotated to one specific pathway divided by all identified metabolites annotated to this pathway. Dot size represents the number of differently abundant metabolites annotated to this pathway (*n* = 6). (**B**) Heatmap constructed by abundances of metabolites involved in the pathway of protein digestion and absorption (*n* = 6).

To analyze functional alterations of pathways in the gut microbiota of mice upon C3G administration, enrichment analysis of the metabolite profile was conducted. Enrichment analysis results indicated that a range of pathways were significantly enriched in the gut microbiota of C3G-treated mice (Figure 6A), such as the PPAR signaling pathway, axon regeneration, mineral absorption, protein digestion and absorption, and so forth. Many pathways of C3G-associated enrichment are involved in the metabolism of amino acids, including protein digestion and absorption, beta-alanine metabolism, phenylalanine, tyrosine and tryptophan biosynthesis, cyanoamino acid metabolism, histidine metabolism, as well as biosynthesis of amino acids. Of particular interest, the pathway of protein digestion and absorption was significantly enriched in the gut microbiome of mice with C3G administration. Figure 6B displayed the distribution of metabolite intensities regarding the pathway of protein digestion and absorption in control and C3G-treated mouse groups. The pattern clearly showed that C3G administration significantly altered intensities of metabolites involved in the pathway of protein digestion and absorption. Levels of multiple amino acids were significantly higher in fecal samples of C3G-treated mice, whereas levels of isobutyric acid were lower in these mice. Given the essential role of the gut microbiota in host fitness, coupled with the health benefits of C3G, alterations of gut microbial metabolites and associated pathways upon C3G intake could contribute to C3G-mediated health effects.

## 4. Discussion

The objective of the present study is to investigate the effects of C3G on the gut microbiota and its metabolite profile. Anthocyanins comprise a variety of molecules, of which C3G is the most classic example of an anthocyanin that is widely present in fruit and vegetables, especially berry fruit. Given the well-recognized health benefits of berries and the essential role of the gut microbiota in various aspects of human health, we conducted metagenomic and metabolomic analyses to explore the impact of C3G on the gut microbiome. The results demonstrated that C3G administration significantly alters the gut microbial composition in mice, leading to changes in a number of gut microbiota-related metabolic products. These findings suggest that C3G not only modulates the gut microbiota at the abundance level, but also alters the metabolite profile of the gut microbiome. Specifically, the protein digestion and absorption pathway was significantly enriched in the gut microbiome of mice upon C3G administration, which may contribute to the health benefits of C3G and berries. Given the low bioavailability of polyphenol compounds [28,29], these findings may provide insights regarding the missing link in mechanisms underlying the health effects of poorly absorbed polyphenols including C3G.

The gut microbiota links dietary structure or functional food components to their health effects on the host. Clarifying the link between the gut microbiota and diet can provide a theoretical basis for dietary modulation of the gut microbiota and, therefore, intervention in related metabolic diseases. Curbing metabolic diseases by modulation of the gut microbiota has become a promising strategy. However, effective approaches to gut microbiota modulation through dietary interventions still need to be explored. Berries or their bioactive components may be a strong candidate. Berries such as black raspberries are recognized to have health benefits, and moderate intake of berries can prevent various human diseases, including diabetes, cardiovascular disease, and cancers [12,13,14,15]. In our previous study, the composition and function of the gut microbiota of healthy mice fed with black raspberries was characterized, and it was found that the gut microbiota composition and metabolic activity of black raspberry-fed mice was significantly different from that of the control group [16]. Furthermore, the black raspberry-fed mice had unique metabolite profiles [17,18], indicating the potential of black raspberries in modulating the gut microbiota and its metabolite profile. Anthocyanins are the main bioactive components of berry fruit, including black raspberries [12,20]. However, due to their strong polarity, anthocyanins are generally considered to not be directly absorbed into the circulatory system of animals or humans, hence resulting in low bioavailability [28,29], suggesting that the gut microbiota and its metabolic activity may play a key role in the health benefits of anthocyanins. The underlying mechanisms remain evasive; however, changes in the gut microbiota and key metabolites may be involved.

To further explore key functional components of black raspberries and the role of the gut microbiota in the health effects of berries, in the present study we selected a typical anthocyanin, cyanidin-3-O-glucoside (C3G), to modulate the gut microbiota of mice, and the gut microbiota composition and metabolite profile were characterized. The results showed that the gut microbiota composition of the C3G group was significantly different from that of the control group and, more importantly, the gut metabolite profile of the C3G group was significantly different from that of the control group. Many characteristic metabolites were identified. The structures of these metabolites are diverse, including bile acids, tocopherols, amino acids and associated derivatives, which are either directly produced or modified by gut bacteria. The gut microbiota participates in host physiology through the production of functional metabolites [30], and the profile of all metabolites in the intestine is referred to as the intestinal metabolome [5]. The microbial composition, gene expression, and metabolic activity in the gut microbiome could be changed via diet or exposure to xenobiotics of the host. Meanwhile, exogenous substances and dietary components were metabolized by various gut bacteria to continually generate diverse metabolites. Some bacterial metabolites serve as crucial signaling molecules, directly or indirectly altering cellular functions and regulating host physiology, meaning they are, consequently, involved in host health and disease [30,31,32]. Thus, profiling and characterizing metabolites of the gut microbiota not only helps to understand the interactions between gut microorganisms and the host from a mechanistic perspective but may also reveal biomarkers closely related to diseases. 

For instance, indole-3-acetic acid (IAA), which belongs to indole-derived metabolites generated by the gut microbiota through tryptophan metabolism, decreased in fecal samples of mice and patients with insulin resistance [33]. IAA can act on the aryl hydrocarbon receptor and increase levels of glucagon-like peptide-1 via regulation of gene expression and cell differentiation [33]. Likewise, bile acids, which are cholesterol derivatives synthesized in liver and can be modified by gut bacteria, are not only involved in digestion and absorption, but also serve as signaling molecules activating nuclear receptors, and are therefore involved in gastrointestinal inflammation and carcinogenesis [32]. In the present study, a number of metabolites were found to be differently abundant in fecal samples of control and C3G groups, including amino acids (e.g., tryptophan), bile acids (e.g., taurocholate), and vitamins (e.g., Vitamin E), which may be associated with effects of C3G. In particular, the pathway of protein digestion and absorption was significantly enriched in the gut microbiome of mice upon C3G administration, suggesting that the metabolic activity of protein metabolism in gut bacteria differed. Specifically, levels of multiple L-amino acids were significantly higher in the fecal samples of C3G-treated mice. A previous study reported that fecal microbiota transplantation improved slow transit constipation [34]. Of interest, the gut microbiota of patients and gut metabolites involved in the protein digestion and absorption pathway were significantly altered, suggesting the role of the protein digestion and absorption pathway in host intestinal functions. In addition, L-amino acids have anti-inflammatory effects on intestinal epithelial cells via the calcium-sensing receptor [35]. In fecal samples of C3G-treated mice, multiple L-amino acids including L-valine, L-glutamine, L-tryptophan, L-histidine, and L-phenylalanine were enriched, therefore probably conferring anti-inflammatory effects. Based on previous research regarding modulation of the gut microbiota by black raspberries, results of this follow-up study further supported the key role of berry component C3G in modulating the gut microbiota by elucidating gut microbiota changes upon C3G administration in mice. Taken together, changes in the gut metabolite profile, especially metabolites involved in the protein digestion and absorption pathway, may be related to the effects of C3G on host health, suggesting that the potential of C3G and other anthocyanins in modulating the gut microbiome warrants future studies.

The findings of the present study indicated that fecal Akkermansia decreased in mice upon C3G administration, in contrast to the significantly increased population of Akkermansia muciniphila in fecal samples of mice fed a black raspberry-rich diet. One possible explanation underlying the conflicted results could be that the boosted Akkermansia muciniphila population in the gut microbiota of mice fed black raspberries was mainly associated with other components instead of C3G. Although polyphenols could be a reason that Akkermansia muciniphila thrives, five anthocyanins besides C3G were present in black raspberries including cyanidin 3-sambubioside, cyanidin 3-xylosylrutinoside, cyanidin 3-rutinoside, and pelargonidin 3-rutinoside [36]. Based on our results, C3G is not responsible for increased fecal levels of Akkermansia muciniphila, which therefore could be due to another anthocyanin. In addition, it is previously reported that berry components other than anthocyanins were also linked with increased Akkermansia muciniphila, such as oligofructose [37]. Taken together, the opposite results regarding changes of Akkermansia muciniphila suggested C3G was not the major component in black raspberries that boosted populations of Akkermansia muciniphila in the gut microbiota, while roles of other anthocyanins or components such as oligofructose warrant further investigation.

Mechanisms underlying changes in the mouse gut microbiota upon C3G administration can be diverse. Since polyphenols are recognized to hardly migrate into the bloodstream, they are thought to act locally in the intestinal tract. On one hand, C3G molecules in the digestive tract could possibly change the intestinal environment that gut bacteria inhabit, including PH values, oxidative stress levels, and concentrations of key metabolites including bile acids, hence affecting metabolic activities and the composition of the residing bacteria. On the other hand, C3G could also possibly impact the host’s intestinal status, including mucus secretion and immune response [38], which in turn influence the gut microbiota. Taken together, possible effects exerted by C3G on the intestinal environment and host cellular functions may be mechanically responsible for alterations in the gut microbiota of mice upon C3G administration. Further studies are needed to clarify the specific mechanism by which C3G causes changes in the gut microbiota.

Admittedly this study was based on the observation of the effects of administration of C3G on the mouse gut microbiota, with no specific mechanism clearly illustrated. However, we profiled and identified metabolic changes in the gut microbiota of mice and identified key metabolic pathways that could contribute to effects of C3G. Although further investigation of mechanisms needs to be pursued, the present study is of significance, depicting the involvement of the gut microbiota in the health effects of anthocyanin C3G.

## 5. Conclusions

In summary, C3G has a significant impact on the gut microbiota composition and key metabolites of mice. This finding is a crucial step toward comprehending how polyphenols affect the gut microbiota and its functions. The results of this study not only provide evidence of the biological activity of natural anthocyanins, but also offer insights for modulating the gut microbiota through bioactive food components. Future research should focus on defining the dose- and time-dependent effects of C3G on the gut microbiota, as well as the effects of other types of anthocyanins on the gut microbiota. Moreover, follow-up studies conducted in human participants would help to validate the findings, and further studies investigating the mechanisms underlying the observed effects are also warranted. Nonetheless, our results indicate that C3G not only changes the gut microbiome at the abundance level, but also substantially alters the metabolic profiles, with changes in key metabolites. Identification of these key metabolites and pathways supports the hypothesis that anthocyanins, including C3G, may lead to health benefits via alterations of the gut microbiota and its metabolic profiles.

## Data Availability

Data is contained within the article.

## Supplementary Materials

The following supporting information can be downloaded at: https://www.mdpi.com/article/10.3390/metabo13070818/s1, Table S1. Significantly altered metabolites in fecal samples of mice upon C3G administration.
